# The Impact of Life History Evolution on the Microbiota of Drosophila Melanogaster

**DOI:** 10.1093/geroni/igaf122.2887

**Published:** 2025-12-31

**Authors:** Nourhan Mahmoud

**Affiliations:** California State University, Fullerton, Downey, California, United States

## Abstract

Manipulation of aging components through laboratory evolution of Drosophila melanogaster offers valuable insights into fast adaptive evolution and has implications for agriculture, conservation, and healthcare. Despite the extensive research on phenotypic and genetic divergence in laboratory populations of D. melanogaster, the role of the microbiome in host adaptation has been largely overlooked. This study examines how life history selection of D. melanogaster impacts its microbiome using the Drosophila Experimental Evolution Population (DEEP) resource. We hypothesize that evolutionary changes in the host phenotype and genotype would correlate with distinct, repeatable patterns in microbiome composition. Four selection regimes, based on age-of-first-reproduction (A-type, 10-day; B-type, 14-day; T-type, 21-day; C-type, 28-day), were compared using metagenomic analysis, colony-forming unit (CFU) plating, and gut dissection. Our results revealed that the microbiomes of each population reflected their evolutionary history, with significant differences in microbial abundance and composition. A-type flies exhibited higher bacterial loads, dominated by lactic and acetic acid bacteria, supporting their faster life history strategy. In contrast, C-type flies, which have a slower life history strategy, exhibited higher Wolbachia abundance, which is known to enhance fecundity and viral resistance. These findings suggest that microbiota composition changes alongside host life history evolution, with certain microbes conferring adaptive benefits that align with the host’s life history strategy. This study highlights the importance of genetic factors in shaping microbiota diversity and underscores the microbiome’s role in host fitness and aging.

